# Enhancing Soybean and Maize Yields through Improved Nitrogen and Soil Water Use Efficiencies: A 40-Year Study on the Impact of Farmyard Manure Amendment in Northeast China

**DOI:** 10.3390/plants13040500

**Published:** 2024-02-10

**Authors:** Yuying Liu, Minsong Bai, Feng Shen, Zhengchao Wu, Jinfeng Yang, Na Li, Houjun Liu, Jian Dai, Xiaori Han

**Affiliations:** 1 Agricultural Resources and Environment Mobile Station, College of Land and Environment, Shenyang Agricultural University, Shenyang 110866, China; liuyuying0325@126.com (Y.L.); bay18111952358@126.com (M.B.); sf123456xx@126.com (F.S.); yangjinfeng7672@syau.edu.cn (J.Y.); lnxlina@163.com (N.L.); liuhoujun_0@syau.edu.cn (H.L.); 2 National Engineering Research Center for Efficient Utilization of Soil and Fertilizer Resources, Shenyang 110866, China; 3 Monitoring & Experimental Station of Corn Nutrition and Fertilization in Northeast Region, Ministry of Agriculture and Rural Affairs, Shenyang 110866, China; 4 Analytical and Testing Center, Shenyang Agricultural University, Shenyang 110866, China; wuzhengchao66@163.com

**Keywords:** manure amendment, soybean–maize–maize rotation, yield, water and N use efficiencies, soil mineral N

## Abstract

The combined application of manure and chemical fertilizers has been recognized as a critical factor driving significant changes in crop yield and nutrient use efficiency, holding the potential to optimize agricultural management to achieve high yields. In this 40-year study, we investigated the effect of manure amendment on soybean and maize yields, water and nitrogen use efficiencies (WUE and NUE), and water and mineral N storage at 0–100 cm soil depths from 2017 to 2018 to explore the optimization of fertilization management strategies for soybean and maize production in Northeast China. To elucidate the impact of chemical fertilizers and manure, twelve treatments—control (CK); single N fertilizer at a low rate (N_1_) and that at a high rate (N_2_); N_1_, phosphorus (P), and potassium (K) fertilizer (N_1_PK); manure alone at 13.5 and 27 t ha^−1^ (M_1_ and M_2_); and those combined with N, P, or K fertilizer (M_1_N_1_, M_1_N_2_, and M_1_N_1_PK and M_2_N_1_, M_2_N_2_, and M_2_N_1_PK)—were selected and studied. The results showed that long-term amendment with manure significantly increased crop biomass and yield in the soybean–maize–maize rotation system. Combining with manure increased the WUE, the partial factor productivity of N fertilizer (PFP_N_), and N physiological efficiency (PE_N_) in both the soybean and maize seasons; conserved soil water (mainly at 40–60 cm); and increased soil N retention (in the upper 60 cm layer), which reduced the risk of N leaching, with a better effect being observed after the application of 13.5 t ha^−1^ manure. These results provide insight into the potential of using fertilization management strategies that include amendment with 13.5 t ha^−1^ manure in combination with N, P, and K fertilizer in the maize season and only chemical fertilizer in the soybean season, as these results indicate that such strategies can achieve high yields and be used to implement agricultural sustainable development in brown soil regions in Northeast China.

## 1. Introduction

Nitrogen (N) plays a vital role in enhancing crop yield by 30–50% [1,2]. However, the overuse of N fertilizer is common, with N fertilizer use greatly exceeding crops’ N requirements. It resulted in no increase, and even a decrease, in crop yield, a low N use efficiency (NUE), N loss, and risks to the environment [3,4,5]. Studies from all over the world have indicated that the average N rate was 74 kg N ha^−1^ yr^−1^ globally, with a NUE of 40% [6]. However, in China, the average NUE was 25%, with a N rate of 305 kg N ha^−1^ yr^−1^ [6]. In Germany, Ehlert et al. [7] showed that the N fertilizer rate could be reduced by 12% without influencing the winter wheat yield. In China, the dual challenge of improving crop yield and reducing environmental risks has received considerable attention [8].

The application of chemical fertilizer combined with manure is an effective way to increase crop yield and improve soil fertility [9,10,11]. In Jilin, China, a long-term experiment showed that the application of N, phosphorus (P), and potassium (K) fertilizers combined with manure significantly increased maize yield by 159 kg yr^−1^ and the soil total N by 0.029 g kg^−1^ yr^−1^ compared to the unfertilized treatment [12]. Quan et al. [13] found that the co-application of chemical fertilizer and manure significantly increased maize yield by 26.6% compared to conventional practices. A 31-year study in Hunan demonstrated that the soil total N concentration in both the rhizosphere and non-rhizosphere was increased by 48–76%, effectively enhancing N accumulation in paddy soil when a portion of N fertilizer was replaced by manure, compared to the sole application of chemical fertilizer [14]. Additionally, after the application of microbial fertilizers combined with manure and soil water, mineral N (NH_4_^+^, NO_3_^−^) increased, as did the crops’ N uptakes, as the full amount of maize straw was returned to the field [15]. Nevertheless, there was no effect on yield increase when over 30% of N fertilizer was replaced by manure due to the slow release of N in manure, which was inadequate for wheat growth [16]. The combined application of N fertilizer and manure can also provide material for denitrifying microorganisms, leading to an increase in N losses by gas [17,18,19]. Therefore, it is essential to investigate manure amendment at high and low application rates.

Additionally, water is a major factor that affects crop yield, especially in rain-fed regions [20,21]. In 68 sites of Southern America, on average, the potential wheat yield was 5200 kg ha^−1^, with a difference of 3000 kg ha^−1^ due to variance in precipitation [22]. In severe drought years, crop yield was decreased dramatically as a result of soil–water imbalances or deficiencies [23,24]. In Jilin, China, maize yield was lower in 2002 and 2009 when the rainfall was less than that in other years, which indicated that maize yield varied with the differed annual precipitation [25]. In the same place, after no-tillage treatment, maize yield was significantly reduced by 29.3% in a dry year (2015) compared to that in a normal year (2013), with a decrease of 200.9% after moldboard plough treatment [13]. Water scarcity has become a global issue limiting crop growth and grain production [26,27,28]. In [29], water use efficiency was improved by 25–40% and 15–25% via soil and nutrient management, respectively.

In Northeast China, an important area with respect to grain production, maize, and soybean are the major cultivated crops, and these crops mainly rely on natural precipitation to meet their water requirements. The uneven distribution of precipitation in this region (mainly occurring between July and August, with little precipitation in the spring) largely influenced the yield formation and changes in N use efficiency, as well as fertilization [6,30]. Therefore, based on a 40-year fertilization field experiment (initiated in 1979) in a brown soil area in Northeast China, we analyzed the grain yield, soil water, and mineral N at 0–100 cm soil depth, as well as the water and N use efficiencies, in 2017 (soybean) and 2018 (maize). The objectives of this study were to (1) evaluate the effect of 40-year manure amendment on soybean and maize grain yields and water and N use efficiencies; (2) investigate water and mineral N storage at 0–100 cm soil depth; and (3) outline an optimized fertilization management strategy to ensure improved grain yields, water and N use efficiencies, and decreased N loss in brown soil areas.

## 2. Materials and Methods

### 2.1. Experimental Site

**Figure 1:**
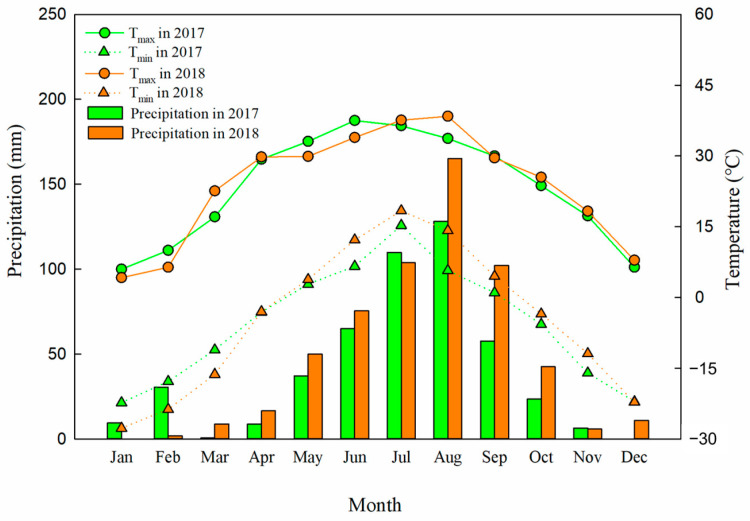
Monthly mean precipitation (mm) and the maximum (T_max_) and minimum (T_min_) temperatures (°C) in the soybean season in 2017 and the maize season in 2018 at the experimental site. Precipitation data were extracted from in the Rain Gauge (adcon) installed near the experimental field. The T_max_ and T_min_ data were provided by the China National Meteorological Administration.

The 40-year fertilization experiment in brown soil, which was ongoing at the time of our study, was initiated in 1979 at the Scientific Research Base of Shenyang Agricultural University, Shenyang, Liaoning Province (40°48′ N, 123°33′ E, altitude 83.6 m). This area is located in the center of the southern area of Songliao Plain, which has a temperate humid-semi-humid monsoon climate. The frost-free period lasts 140–180 days. Details regarding monthly temperature and precipitation for the soybean season in 2017 and the maize season in 2018 are presented in Figure 1, with the average maximum and minimum temperature and annual rainfall being 38.0 °C, −25.0 °C, and 530 mm. According to the United Nations FAO classification, the soil used was haplic luvisol [31]. The physicochemical properties of the original soil (0–20 cm) in 1979 were as follows: 105.5 mg N kg^−1^ alkaline hydrolysable N, 6.50 mg P kg^−1^ available P, 97.9 mg K kg^−1^ available K, 0.80 g N kg^−1^ total N, 0.38 g P kg^−1^ total P, 21.1 g K kg^−1^ total K, 15.9 g kg^−1^ organic matter, and a pH of 6.50.

### 2.2. Experimental Design and Field Management

The long-term experiment was arranged in a randomized block design, as described by Li et al. [10]. Twelve treatments in the field experiment were selected in this study: control (CK); single N fertilizer at a low rate (N_1_) and that at a high rate (N_2_); N_1_ combined with phosphorus (P) and potassium (K) fertilizer (N_1_PK); the single application of manure at 13.5 t ha^−1^ (M_1_); M_1_ combined with N, P, or K fertilizer (M_1_N_1_, M_1_N_2_, and M_1_N_1_PK); the single application of manure at 27 t ha^−1^ (M_2_); and M_2_ combined with N, P, or K fertilizer (M_2_N_1_, M_2_N_2_, and M_2_N_1_PK). The N, P, and K fertilizers were applied as urea, superphosphate, and potassium sulfate, respectively, and the organic fertilizer was pig manure. The average nutrient concentrations of the pig manure from the past 40 years were as follows: 83.5 g kg^−1^ organic carbon, 8.7 g kg^−1^ P_2_O_5_, and 10.0 g kg^−1^ K_2_O [10,11]. Table 1 shows the application rates of chemical and organic fertilizers in the soybean and maize seasons. All the fertilizers were supplied as basal fertilizer before sowing and then incorporated into the 0–20 cm topsoil using a rotavator.

In late April 2017, a local soybean cultivar, Liaodou 15, was sown in each plot (16 × 10 m) at a seeding rate of 150,000 plants ha^−1^, with 11 cm wide plant spacing and a ridge width of 60 cm. In late April 2018, “Dongdan 6531”, a widely used local maize cultivar, was sown at a seeding rate of 60,000 plants ha^−1^, with 27 cm plant spacing and a ridge width of 60 cm. The soybean and maize were both harvested in early October, and then, the straw was moved away while the stubble was left in the field. There was no additional irrigation except for natural rainfall, and the field management strategies adopted were similar to those of local farmers, including for weed and pest control.

### 2.3. Sampling and Chemical Analyses

#### 2.3.1. Soil

Soil samples from five duplicate cores were evenly collected on the ridge platform from each plot to a depth of 100 cm with an interval of 20 cm at soybean and maize sowing and harvest in 2017 and 2018. Soil samples from the same plot were mixed and sealed for the subsequent measurement of the soil water and mineral N content. The soil water content was determined by using the drying method (105 °C for 24 h). The soil mineral N was extracted with 50 mL of 1 mol L^−1^ KCl (1:10 soil:solution) and analyzed using a continuous flow analyzer (AA3, SEAL Analytical, Norderstedt, Germany).

#### 2.3.2. Soybean and Maize

At soybean maturity (early October in 2017), plants covering two ridges in a length of 2 m were selected randomly and harvested from three 6 × 5 m areas in each plot. The plants were cut off at the point of root and stem connection using sickles and then put into net bags for air-drying. The air-dried soybean plants were weighed, threshed, and separated into grains, pods, and straw (including the stems and leaves) to determine the biomass and N uptake. The air-dried subsamples were oven-dried at 90 °C for 0.5 h and then at 65 °C for 48 h to calculate the water content and dry weight before being ground and passed through a 0.5 mm sieve and then digested using H_2_SO_4_-H_2_O_2_ for the determination of the total N. The plant N concentration was also analyzed using a continuous flow analyzer. Similarly, maize plants covering three ridges in a length of 2 m were collected and partitioned into straw (including the stems and leaves), bracts, cobs, and grains and weighed as described above before being subjected to subsample analysis.

### 2.4. Data Calculations

The aboveground N uptake of the soybean and maize crops was calculated as follows:

Aboveground N uptake (kg N ha^−1^) = ∑(N concentration in each part × dry weight)/1000 (1)

where the N concentration in each part (g N kg^−1^) refers to that of soybean grains, pods, and straw or maize straw, bracts, cobs, and grains; dry weight is the biomass of each soybean or maize organ (kg ha^−1^); and 1000 is the conversion coefficient.

The partial factor productivity of the N fertilizer (PFP_N_, kg kg^−1^) was expressed as [32]

PFP_N_ (kg kg^−1^) = GY/N rate (2)

where GY (kg ha^−1^) is the grain yield, and N rate (kg N ha^−1^) is the N fertilizer application rate.

The N physiological efficiency (PE_N_, kg kg^−1^) was defined as grain yield divided by the total crop N uptake [33,34], which was expressed using the following equation:

PE_N_ (kg kg^−1^) = GY/Aboveground N uptake (3)

The soil water storage (SWS, mm) at 0–100 cm depth was calculated in accordance with Zhang et al. [35]:

(4) SWS (mm)=∑SWC ×BD×SD/10

where SWC (%) is the water content in the corresponding soil layer.

Surface runoff and drainage were ignored in this study since the field used in the field experiment was flat and the upward flow of groundwater reaching the root zone was negligible. Therefore, natural rainfall was the only water source during the crop growing seasons. The evapotranspiration (ET, mm) was calculated using the soil water balance equation (also used by Zhang et al. [35] and Li et al. [36]):

ET (mm) = P + ∆SWS(5)

where P is the precipitation (mm) during the soybean or maize growing seasons, obtained from a Rain Gauge (adcon) installed in the experimental field, and ∆SWS (mm) is the variation in soil water storage at 0–100 cm soil depth between soybean or maize sowing and harvest.

The soybean and maize water use efficiencies (WUE, kg ha^−1^ mm^−1^) were calculated as follows [36]:

WUE (kg ha^−1^ mm^−1^) = GY/ET (6)

The amount of NH_4_+ and NO_3_^−^ in each soil layer was calculated as by Dai et al. [3]:

(7) $${\mathrm{NH}}_{4}+/{{\mathrm{NO}}_{3}}^{-}\;(\mathrm{kg}\;N\;{\mathrm{ha}}^{-1})=\frac{SD\times BD\times C_{i}}{10}$$

where SD (cm) is the soil depth, BD (g cm^−3^) is the soil bulk density, C_i_ is the NH_4_^+^ or NO_3_^−^ concentration (mg N kg^−1^) in the corresponding soil layer, and 10 is the conversion coefficient.

The amount of mineral N (kg N ha^−1^) was calculated as NH_4_^+^ plus NO_3_^−^.

### 2.5. Statistical Analysis

SPSS 21.0 (Systat Software, Inc., Chicago, IL, USA) was used for statistical analysis. An ANOVA with a least significant difference test at the 0.05 probability level (*p* < 0.05) was performed to analyze the significant differences in grain yield, biomass, PFP_N_, PE_N_, water use efficiency, and other measures.

## 3. Results

### 3.1. Soybean and Maize Biomass and Yields

**Figure 2:**
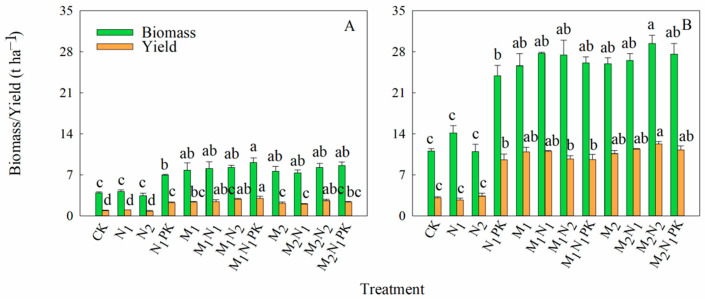
Biomass and grain yields of soybeans in 2017 (**A**) and maize in 2018 (**B**). Different lowercase letters indicate significant differences in the biomass and yield among treatments (*p* < 0.05).

The soybean and maize biomass and grain yields varied dramatically with long-term fertilization (Figure 2). The application of the N, P, and K fertilizer (N_1_PK) significantly increased soybean and maize biomass and yields compared to the CK and N alone treatments, while the combination with manure consistently enhanced the biomass and yields. In 2017 (Figure 2A), after treatment with 13.5 t ha^−1^ manure, the soybean biomass increased by 91.3% compared to the soybean crops treated with chemical fertilizer alone, with the application of 9.11 t ha^−1^ M_1_N_1_PK fertilizer achieving the greatest increase, which was 30.8% greater than that achieved with the application of N_1_PK. Regarding soybean yield, it increased by 146.9%, with the greatest soybean yield, which was 34.6% higher than that achieved with N_1_PK, achieved using 2.98 t ha^−1^ M_1_N_1_PK. Upon combination with manure at 27 t ha^−1^, the soybean biomass and yield increased by 83.7% and 116.7%, respectively, compared to those achieved with solely chemical fertilizer, where the highest biomass was achieved with 8.59 t ha^−1^ M_2_N_1_PK. In 2018 (Figure 2B), compared to those treated with chemical fertilizer alone, the combination with 13.5 t ha^−1^ manure increased the maize biomass and yield by 96.6% and 187.3%; increases in the maize biomass and yield of 101.3% and 211.7% were achieved after the addition of 27 t ha^−1^ manure, though no significant differences among the manure treatments were found. Additionally, after the application of M_1_N_1_PK and M_2_N_1_PK, the maize biomass and yield increased by 9.2% and 0.5% and 15.6% and 17.8%, respectively, compared to the N_1_PK treatment, though no significant differences in the maize biomass and yield were found (*p* < 0.05).

### 3.2. Soil Water Storage in the Soybean Season

**Figure 3:**
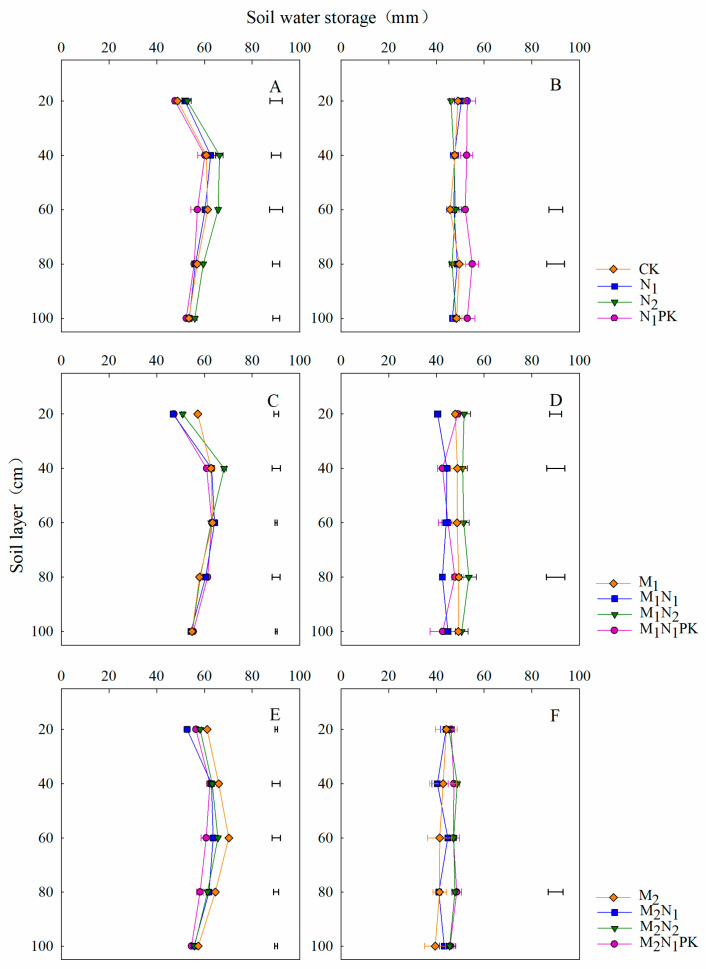
Soil water distributions in the 0–100 cm soil layer before soybean sowing (**A**,**C**,**E**) and after harvest (**B**,**D**,**F**) in 2017. The horizontal lines indicate the least significant difference (LSD) values among the treatments (*p* < 0.05) in the same soil layer.

The soil water distributions at 0–100 cm depth before soybean sowing differed with those after harvest (Figure 3). Before soybean sowing, soil water was mainly distributed in the 40–80 cm soil layer, and a similar trend was found for all treatments (Figure 3A,C,E). The water storage values at 0–100 cm soil depth for the treatments involving the application of chemical fertilizer alone (CK, N_1_, N_2_, and N_1_PK) were 281, 285, 300, and 273 mm, respectively (Figure S1), with soil water mainly accumulating at 40–80 cm soil depth, while water storage values of 296, 289, 295, and 288 mm were found for the treatments including 13.5 t ha^−1^ manure, with soil water mainly accumulating at 40–60 cm soil depth, and water storage values of 319, 297, 304, and 292 mm were found for the treatments involving 27 t ha^−1^ manure, with soil water accumulation mainly occurring in the 40–80 cm soil layer. At soybean harvest (Figure 3B,D,F), the water storage at 0–100 cm soil depth decreased by 13.4% on average for the treatments involving only a single chemical fertilizer, while values of 19.0% and 26.2% were recorded for treatment with manure at 13.5 and 27 t ha^−1^, respectively, compared to that before soybean sowing. The soil water was distributed evenly in the 0–100 cm soil layer for all treatments.

### 3.3. Soil Water Storage in the Maize Season

**Figure 4:**
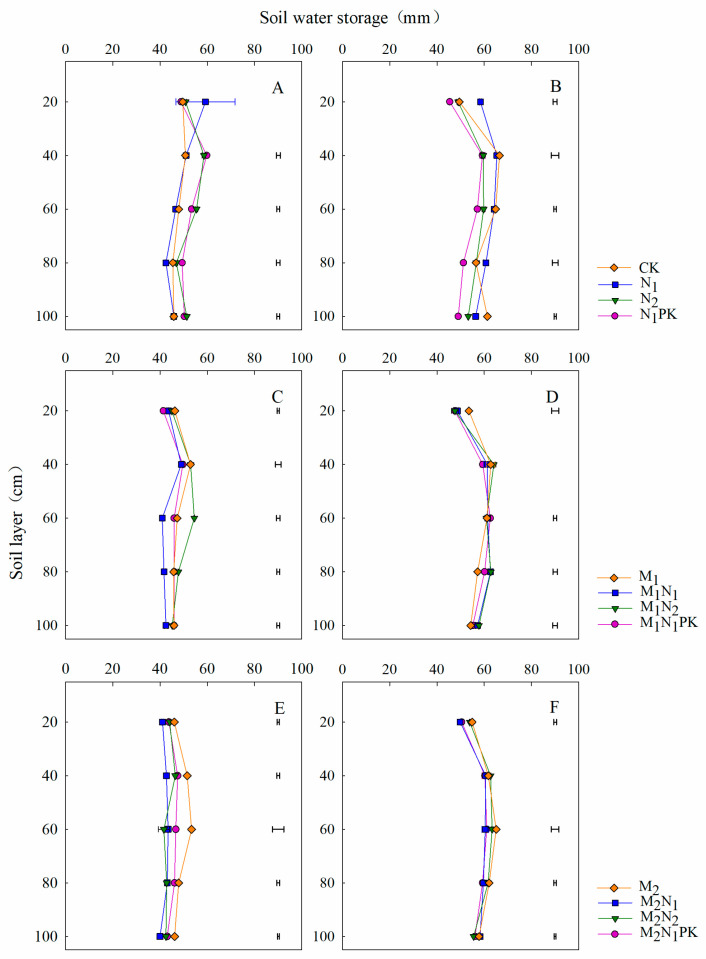
Soil water distributions at 0–100 cm soil depth before maize sowing (**A**,**C**,**E**) and after harvest (**B**,**D**,**F**) in 2018. The horizontal lines indicate the least significant difference (LSD) values among the treatments (*p* < 0.05) in the same soil layer.

Before maize sowing, the soil water distribution at 0–100 cm depth was similar, but significant differences among the treatments were found (Figure 4A,C,E). For the chemical fertilizer-only treatments, the soil water was distributed evenly in the 0–100 cm layer, where it mainly occurred at 40–80 cm depth for the manure treatments. After maize harvest, the soil water storage in the 0–100 cm layer increased, on average, by 13.9%, 24.8%, and 31.0% for the treatments involving solely chemical fertilizer in combination with 13.5 or 27 t ha^−1^ manure, respectively, compared to that before sowing (Figure 4B,D,F). For the treatments involving the application of a chemical fertilizer only, soil water was distributed evenly in the 0–100 cm soil layer, and the lowest storage value of 262 mm belonged to N_1_PK (Figure S2). There was no significant difference in soil water storage at 0–100 cm depth among the treatments including both 13.5 and 27 t ha^−1^ manure, with soil water mainly accumulating in the 40–80 cm layer.

### 3.4. Soil Mineral N Accumulation in the Soybean Season

**Figure 5:**
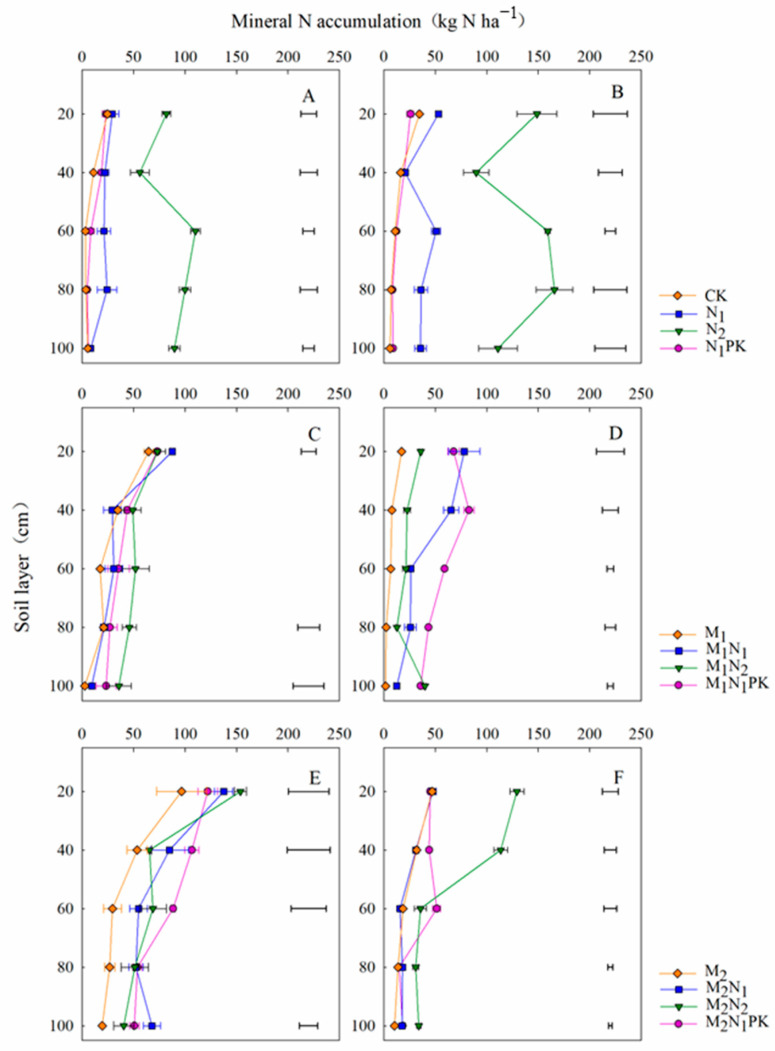
Soil mineral N distribution at 0–100 cm depth at soybean sowing (**A**,**C**,**E**) and harvest (**B**,**D**,**F**) in 2017. The horizontal lines indicate the least significant difference (LSD) values among the treatments (*p* < 0.05) in the same soil layer.

The mineral N accumulated in the 0–100 cm soil layer was measured in 2017 (the soybean season) after 39 years of fertilization. At soybean sowing, the mineral N accumulated at 0–100 cm soil depth was 105 and 438 kg N ha^−1^ for N_1_ and N_2_, significantly higher than the values for CK and N_1_PK (Figure 5A and Figure S3), where the residual N peaks of 29.2 and 110.1 kg N ha^−1^ occurred in the 20 and 60 cm layers, respectively. Soil mineral residual N at 0–100 cm depth increased as the manure rate increased and mainly accumulated at 0–60 cm depth with the application of manure both at 13.5 and 27 t ha^−1^, while it decreased in the 80–100 cm layer (Figure 5C,E). At soybean harvest and at 0–100 cm depth, with chemical fertilizer alone, soil mineral N increased to 91 (N_1_) and 237 kg N ha^−1^ (N_2_), values higher than those at pre-sowing, with the residual peaks of 53.2 and 165.8 kg N ha^−1^ occurring at depths of 20 and 80 cm (Figure 5B and Figure S3). With the application of chemical fertilizer in concert with 13.5 t ha^−1^ manure (Figure S3), the soil residual N at 0–100 cm depth was significantly higher than that achieved with the application M_1_, while the greatest value (284 kg N ha^−1^) belonged to M_1_N_1_PK, peaking (82.7 kg N ha^−1^) at 40 cm soil depth and decreasing at 40–100 cm depth. Regarding the treatments with 27 t ha^−1^ manure, the soil mineral N mainly accumulated at 0–60 cm depth, while it decreased in the 80–100 cm soil layer. The residual mineral N in the 0–100 cm layer was the highest (344 kg N ha^−1^) with the M_2_N_2_ treatment, followed by the M_2_N_1_PK treatment, with the residual peak (51.5 kg N ha^−1^) being recorded at 60 cm soil depth.

### 3.5. Soil Mineral N Accumulation in the Maize Season

**Figure 6:**
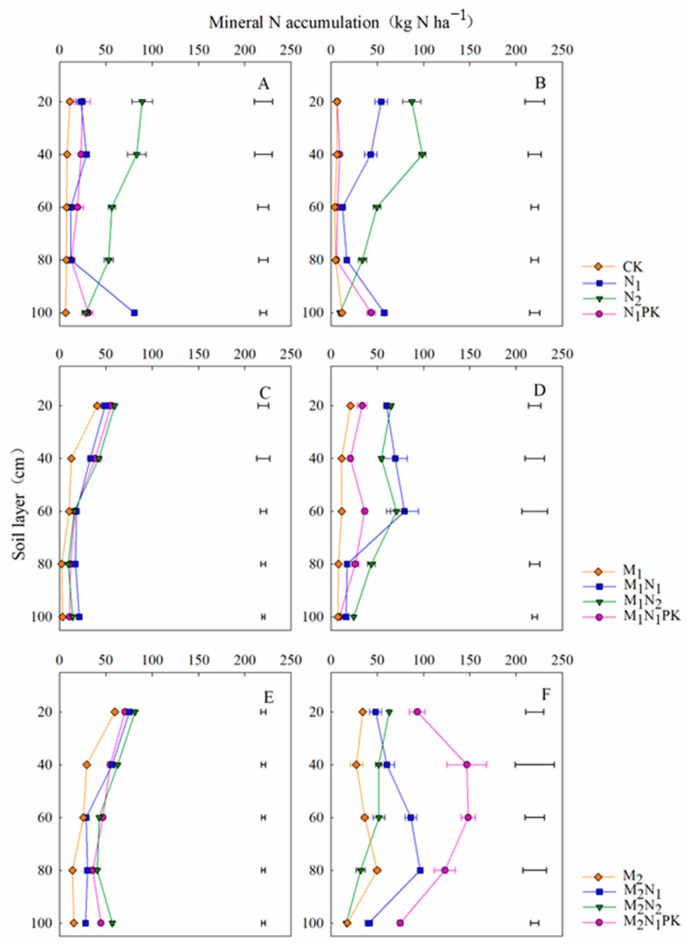
Soil mineral N distribution at 0–100 cm depth at maize sowing (**A**,**C**,**E**) and harvest (**B**,**D**,**F**) in 2018. The horizontal lines indicate the least significant difference (LSD) values among treatments (*p* < 0.05) in the same soil layer.

Before maize sowing in 2018, the soil mineral N values at 0–100 cm depth achieved with the use of chemical fertilizer alone (except for N_2_) were higher than those achieved with the use of 13.5 t ha^−1^ manure. However, after combining with 27 t ha^−1^ manure, the soil mineral N values at 0–100 cm depth were increased compared to those achieved with chemical fertilizer used in isolation (Figure 6A,C,E and Figure S3). At maize harvest after the 40-year application of chemical fertilizer (Figure 6B), soil mineral N was mainly distributed in the 0–40 cm and 80–100 cm layers. The soil mineral N accumulated in the 0–100 cm layer with N_2_ was significantly higher than that with other treatments, with a peak of 98.7 kg N ha^−1^ at 40 cm soil depth, and the mineral N with N_1_PK was significantly lower than that with N_1_ and N_2_ (Figure 6B and Figure S4). The combined application of manure both at 13.5 and 27 t ha^−1^ increased the soil mineral N in the 0–100 cm layer compared to that before sowing (Figure 6D,E), with the soil mineral N mainly distributed at 0–80 cm soil depth, with a significant decrease occurring in the 80–100 cm layer. The soil mineral N in the 0–100 cm layer was high with 27 t ha^−1^ manure and increased by 287.5% and 140.2%, respectively, compared to those with chemical fertilizer alone and 13.5 t ha^−1^ manure, where the largest value (586 kg N ha^−1^) was found to belong to the M_2_N_1_PK treatment (Figure S4).

### 3.6. N and Water use Efficiencies in the Soybean and Maize Seasons

Manure amendment significantly increased the PFP_N_ compared to the CK and chemical fertilizer alone treatments in the soybean season, with the highest value found for the M_1_N_1_PK fertilizer (99.3 kg kg^−1^) being 34.6% and 27.1% higher than those derived from the application of N_1_PK and M_2_N_1_PK (Table 2). Long-term fertilization with 13.5 t ha^−1^ manure increased the PE_N_ by 6.3% and 53.8%, respectively, compared to the use of solely chemical fertilizer and 27 t ha^−1^ manure. The PE_N_ decreased as the manure rate increased, and it was the lowest (8.3 kg kg^−1^) with the application of M_2_N_1_PK. Regarding the treatments with chemical fertilizer alone, the greatest WUE value of 5.5 kg ha^−1^ mm^−1^ was found for N_1_PK (significant difference). For the treatments combined with 13.5 and 27 t ha^−1^ manure, the WUEs increased by 143.2% and 101.1% on average, respectively, compared to the unfertilized and single chemical fertilizer treatments, with the highest (6.5 kg ha^−1^ mm^−1^) value being found for M_1_N_1_PK (Table 2). In the maize season, combining with manure significantly increased the PFP_N_ compared to the use of chemical fertilizer alone. The PFP_N_ increased as the manure rate increased by 106.4% and 13.1% on average with the application of 27 t ha^−1^ manure, respectively, compared to the application of chemical fertilizer in isolation and 13.5 t ha^−1^ manure. The PE_N_ values derived from the treatments with 13.5 and 27 t ha^−1^ manure improved by 10.7% and 20.5% on average, respectively, relative to those achieved with chemical fertilizer alone. The addition of manure significantly increased the WUE, but there were no significant differences among the treatments, with values of 21.8 and 25.8 kg ha^−1^ mm^−1^ found for M_1_NPK and M_2_NPK, respectively.

## 4. Discussion

### 4.1. Effect of Manure Amendment on the Soybean and Maize Biomass and Yields

The amendment with farmyard manure increased the crops’ ability to absorb and use nutrients and water, and nutrient absorption and use play an important role in regulating crop growth and development. Farmyard manure amendment also improved the crop yield and nutrient use efficiency [10,13,37]. Forty-year fertilization significantly increased the soybean and maize yields compared to the no fertilizer treatment, and yield was not affected by the N rate (Figure 2), which was mainly due to the sufficient N supply [38]. This is consistent with results indicating that fertilization enhanced the maize yield dramatically compared to the non-fertilization treatment, but no significant differences between the treatments with different N application rates were found [35]. In our study, which included an amendment with pig manure for 40 years, the highest biomass and yield values were achieved by M_1_N_1_PK in the soybean season and M_2_N_2_ in the maize season, with values of 29.407 and 12.271 t ha^−1^, respectively (Figure 2). Similarly, Fan et al. [39] found that the application of manure for 35 years increased the maize yield and soil N accumulation, with these changes attributed to the enhancement of the embedded rock microbe group abundance, which was positively correlated with soil function and crop yield [13]. The application of manure, especially in combination with chemical fertilizer, is a carbon sink process in the soil carbon–nutrient cycle that is beneficial for improving the structure and physical and chemical properties of soil, increasing the soil organic matter content, preserving soil fertility and water, and, thus, promoting crop growth and development. In Jilin, Northeast China, the amendment with 4.5 Mg ha^−1^ pig manure for 25 years increased the soil organic matter in the 0–20 cm soil layer [40]. In our study, the application of chemical fertilizers in conjunction with manure fertilized the soil and provided a sufficient supply of nutrients for crop growth. Therefore, the combination of manure and chemical fertilizers should be adopted to provide nutrients for crop growth and increase biomass and yields in brown soil areas in Northeast China.

### 4.2. Effect of Manure Amendment on Soil Water and N Distributions

Soil water distribution in the 0–100 cm soil layer differed with the different fertilization management strategies (Figure 3 and Figure 4). At soybean and maize sowing, soil water was mainly stored in the 40–80 cm layer with chemical fertilizer alone and in the 40–60 cm layer with 13.5 and 27 t ha^−1^ manure, effectively retaining water and alleviating the water demand during the sowing period, thereby promoting seed germination and emergence and providing a foundation for high-yield formation [41,42]. Zhao et al. [43] indicated that the winter wheat grain yield was linearly correlated with the available soil water or that in the soil profile at planting. In our study, soil water in the 0–100 cm layer did not change significantly from sowing to harvest, potentially due to the seasonal high precipitation, and was mainly concentrated during the soybean and maize growing seasons (Figure 1). In the maize season, the soil water content and storage at 0–100 cm depth were both low with the application of N_1_PK compared to treatment with CK, N_1_, and N_2_ at harvest (Figure 4B and Figure S2). The main reason for this was that the maize yield was significantly increased with the application of N_1_PK, which consumed a great amount of water due to soil evaporation and crop utilization. This is consistent with the results indicating that the differences in soil moisture content among the different treatments gradually increased at each growth stage during the maize growing season [35].

Yields are related to the N demand [44]. However, excessive N application could lead to soil nitrate N accumulation, promoting nitrate N leaching in the deeper soil layers, making the use of nutrients more difficult for crops [45,46,47]. The results of our previous study, which involved ^15^N tracing in the same experiment, indicated that the N use efficiency during the soybean season was only 28.8% [11] and that during the maize season was lower, standing at 12.6% (unpublished data). This not only increased N loss but also resulted in adverse effects such as nitrate groundwater contamination and soil acidification [48,49]. Manure amendment reduced the mineral N input and increased the soil N storage, thus decreasing the N loss to the environment [12]. A previous study showed that approximately 45% of the N absorbed by wheat was from the applied N, while the remaining 55% was from the soil N [50]. The accumulation of nitrate N in the 0–100 cm soil layer can quantitatively reflect the soil N supply capacity [51]. In our study, the soil mineral N tended to accumulate in the upper 60 cm layer with the manure amendment, which was conducive to increasing the N retention, reducing N accumulation in the deeper soil layers, and, therefore, reducing the risk of mineral N leaching (Figure 5 and Figure 6). Duan et al. [52] also proposed that the combined application of manure and reduced chemical fertilizer minimized N loss by leaching, which confirmed the improvement of N storage by manure amendment. However, some studies have shown that the unreasonable application of manure caused a large accumulation of nitrate N and increased the risk of N loss [53], which is consistent with our study, i.e., N, P, and K fertilizer combined with 27 t ha^−1^ manure increased the mineral residual N in the 0–100 cm layer, which increased the risk of N leaching (Figure S4).

### 4.3. Effect of Manure Amendment on Water and N Use Efficiencies

Optimizing the nutrient input potentially reduces the risk of soil water depletion [35]. Fertilization promoted canopy growth and water utilization by stimulating transpiration and reducing the ratio of evaporation to transpiration, thereby increasing the rates of photosynthesis and transpiration and enhancing crop yields [54,55]. Zhang et al. [35] confirmed that N fertilizer application increased the wheat yield significantly and water use efficiency (WUE) over the course of a 10-year experiment. In our study, the combination of chemical and organic fertilizers improved the WUE compared to the use of chemical fertilizer alone, and a similar trend was observed for the yields in both the soybean and maize seasons. In the soybean season (2017), the WUE was at its highest after the application of 13.5 t ha^−1^ manure, standing at 5.83 kg ha^−1^ mm^−1^ on average, while it was 26.68 kg ha^−1^ mm^−1^ with 27 t ha^−1^ manure in the maize season (2018). Improving the WUE promoted the nitrification of ammonium N and the diffusion and flow of nutrients, especially enhanced N assimilation, and ultimately increased the crop yield [42,56]. Similarly, the combination of chemical fertilizer and 13.5 t ha^−1^ manure in the soybean season and 27 t ha^−1^ in the maize season resulted in higher yields (Figure 2).

Temperature and seasonal precipitation affected the soil biological activity and impacted the yield and N use efficiency (NUE) [57,58]. The partial factor productivity of N fertilizer (PFP_N_) demonstrates the capability of the applied N to produce the grain yield [32,59]. The N physiological efficiency (PE_N_) reflects a crop’s capacity to produce grain with the N that has accumulated within it [33]. Fertilization management significantly influences the PFP_N_ and PE_N_ (Table 2). In a two-year field experiment in Denmark, it was found that the PFP_N_ decreased from 45 to 15 kg kg^−1^ as the N rate increased from 85 to 250 kg N ha^−1^ [60]. The PFP_N_ of wheat could reach 41–50 kg kg^−1^ under optimized N conditions [61,62]. In Southern Spain, PE_N_ values of 36.0, 35.0, 33.2, and 32.2 kg kg^−1^ at N rates of 0, 50, 100, and 150 kg N ha^−1^, respectively, were recorded [63]. Manure amendment clearly increased the PFP_N_ compared to the use of chemical fertilizer alone and the control treatment. In the soybean season, the application of 13.5 t ha^−1^ manure had the best effect in terms of improving the PFP_N_, while 27 t ha^−1^ manure presented the highest PFP_N_ in the maize season. Similar to the WUE and PFP_N_, the manure amendment showed a numerically higher PE_N_, which, in turn, increased the crop yield (Table 2). Duncan et al. [64] also reported a similar increase in NUE and found that these responses might be caused by increases in the crop biomass and yield.

### 4.4. Evaluation for Fertilization Recommendation

**Figure 7:**
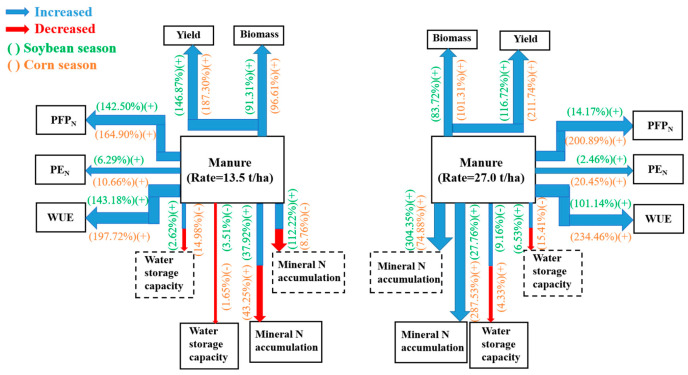
Effects of the combined application of chemical fertilizer and 13.5 and 27 t ha^−1^ of manure on the biomass, yields, nitrogen utilization, and water utilization of soybean and maize crops. The data near each arrow indicate the effects of applying 13.5 and 27 t ha^−1^ manure on the soybean and maize biomass, yield, PFP_N_, PE_N_, WUE, soil N accumulation, and water storage, which are indicated by the arrows (data are the average values after application with 13.5 and 27 t ha^−1^ manure compared to only the use of chemical fertilizer). + indicates a positive effect, while − indicates a negative effect. The thickness of the arrows represents the strength of the relationship between the two variables (average values for soybean and maize). The solid lines show the soil water and mineral N storage at harvest, while the dashed lines indicate the soil water and mineral N storage at sowing.

Manure amendment with reduced chemical fertilizer is an effective nutrient management strategy for improving soil fertility and promoting sustainable crop production [12]. The reasonable application of manure is beneficial for improving soil nutrient use, crop yields, and water and nutrient use efficiencies, meaning that this strategy has considerable potential implications for soybean and maize production (Figure 7). In the studied brown soil area in Northeast China, manure amendment with chemical fertilizer increased the crop biomass and yield and improved the NUE (PFP_N_ and PE_N_) and WUE. Amendment with manure at both 13.5 and 27 t ha^−1^ led to positive effects on the soybean and maize biomass, yield, WUE, and NUE, but the higher manure rate of 27 t ha^−1^ increased the mineral N accumulation in the 0–100 cm soil layer, which enhanced the risk of N leaching into deeper soil depth and increased the consumption of manure resources. Additionally, the combined application of N, P, K, and manure (M_1_N_1_PK) performed better in terms of soybean and maize production, water and N use efficiency, and soil N retention. Therefore, the application of 13.5 t ha^−1^ manure in combination with chemical fertilizer in the soybean and maize rotation system (i.e., manure and chemical fertilizer in the maize season, only chemical fertilizer in the soybean season) is a recommended fertilization management strategy for brown soil areas in Northeast China.

## 5. Conclusions

Amendment with farmyard manure significantly increased the crop biomass and yield in the soybean–maize–maize rotation system. The results indicate that the highest values regarding soybean biomass and yield were achieved with the use of M_1_N_1_PK, with these values being 9.11 and 2.98 t ha^−1^, respectively. In the maize season, on average, treatment with 13.5 and 27 t ha^−1^ manure increased the biomass and yield by 96.6%, 187.3%, 101.3%, and 211.7%, respectively, compared to the use of chemical fertilizer alone. Before soybean and maize sowing, the amendment with manure led to enhanced soil water conservation, with the soil water mainly being stored in the upper 40–60 cm soil layer, compared to that with only the use of chemical fertilizer. The addition of manure increased the N retention, and N tended to accumulate in the upper 60 cm layer, which reduced the risk of N leaching. The WUE, PFP_N_, and PE_N_ values at the treatments involving the combined application of chemical fertilizer and manure were significantly higher than those derived from the no fertilizer and single chemical fertilizer treatments. In the soybean season, the amendment with 13.5 t ha^−1^ manure dramatically increased the WUE and PFP_N_, with the greatest values of 6.52 kg ha^−1^ mm^−1^ and 99.31 kg kg^−1^ achieved by M_1_N_1_PK. In the maize season, the addition of 27 t ha^−1^ manure led to the best improvement in maize production and soil N accumulation but increased the risk of N leaching. In conclusion, the amendment with manure at 13.5 t ha^−1^ in combination with the N, P, and K fertilizers in the maize season and only chemical fertilizer in the soybean season is a recommended fertilization management strategy that can be used to improve the utilization of water and N and, thus, crop production in the soybean and maize rotation system in brown soil areas in Northeast China. In the future, we may conduct further research using tracing techniques (i.e., ^18^O and ^15^N tracing) to specifically quantify the water and N use efficiency.

## Figures and Tables

**Table 1:** The chemical and organic fertilizer rates in the soybean and maize seasons in the 40-year experiment.

Treatments	Chemical Fertilizer Rates (kg ha^–1^)						Pig Manure (t ha^–1^)	
	N		P_2_O_5_		K_2_O			
	Soybean	Maize	Soybean	Maize	Soybean	Maize	Soybean *	Maize
CK	0	0	0	0	0	0	0	0
N_1_	30	120	0	0	0	0	0	0
N_2_	60	180	0	0	0	0	0	0
N_1_PK	30	120	90	60	90	60	0	0
M_1_	0	0	0	0	0	0	13.5	13.5
M_1_N_1_	30	120	0	0	0	0	13.5	13.5
M_1_N_2_	60	180	0	0	0	0	13.5	13.5
M_1_N_1_PK	30	120	90	60	90	60	13.5	13.5
M_2_	0	0	0	0	0	0	27	27
M_2_N_1_	30	120	0	0	0	0	27	27
M_2_N_2_	60	180	0	0	0	0	27	27
M_2_N_1_PK	30	120	90	60	90	60	27	27

CK: Control; N_1_: single N fertilizer at a low rate; N_2_: single N fertilizer at a high rate; N_1_PK: N_1_ combined with P and K fertilizer; M_1_: single application of manure at 13.5 t ha^−1^; M_1_N_1_, M_1_N_2_, and M_1_N_1_PK: M_1_ combined with chemical N, P, or K fertilizer; M_2_: single application of manure at 27 t ha^−1^; M_2_N_1_, M_2_N_2_, and M_2_N_1_PK: M_2_ combined with N, P, or K fertilizer. * Pig manure was not applied from 1992 onwards in the soybean season and applied normally in the maize season.

**Table 2:** PE_N_, PFP_N_, and WUE values in the soybean season (2017) and maize season (2018).

Year		Treatment											
		CK	N_1_	N_2_	N_1_PK	M_1_	M_1_N_1_	M_1_N_2_	M_1_N_1_PK	M_2_	M_2_N_1_	M_2_N_2_	M_2_N_1_PK
2017	PFP_N_^a^	--	34.14c	13.43d	73.80b	--	80.17b	47.13c	99.31a	--	67.44b	43.04c	78.16b
	PE_N_^b^	13.08a	8.67de	8.92cde	9.09cde	10.98b	9.95bcd	10.95b	9.42cde	10.24bc	9.25cde	10.11bc	8.34e
	WUE ^c^	2.04c	2.26c	1.75c	5.46ab	5.17ab	5.12ab	6.51a	6.52a	4.22b	4.20b	5.54ab	5.16ab
2018	PFP_N_	--	90.06d	56.38d	318.82b	--	366.80ab	161.76c	320.54b	--	380.17a	204.52c	375.56a
	PE_N_	46.22b	24.65e	36.99c	37.08c	51.18a	39.93c	31.17d	31.76d	38.02c	47.08b	38.21c	39.03c
	WUE	7.05e	6.17e	7.03b	19.29d	24.54bc	26.02ab	21.63cd	21.83cd	24.14bc	27.30ab	29.45a	25.83ab

^a^ Partial factor productivity of N fertilizer (kg kg^−1^); ^b^ N physiological efficiency (kg kg^−1^); ^c^ water use efficiency (kg ha^−1^ mm^−1^). Different lowercase letters indicate significant differences among treatments at *p* < 0.05.

## Data Availability

The data presented in this study are available on request from the corresponding author.

## Supplementary Materials

The following supporting information can be downloaded at: https://www.mdpi.com/article/10.3390/plants13040500/s1, Figure S1: Soil water storage in the 0–100 cm soil layer before soybean sowing and after harvest in 2017. Different lowercase letters indicate significant differences in soil water storage in the 0–100 cm layers among treatments at *p* < 0.05. Figure S2: Soil water storage in the 0–100 cm soil layer before maize sowing and after harvest in 2018. Different lowercase letters indicate significant differences in soil water storage in the 0–100 cm layers among treatments at *p* < 0.05. Figure S3: Mineral N residual in the 0–100 cm soil layer before soybean sowing and after harvest in 2017. Different lowercase letters indicate significant differences in soil mineral N residual among treatments at *p* < 0.05. Figure S4: Mineral N residual in the 0–100 cm soil layer before maize sowing and after harvest in 2018. Different lowercase letters indicate significant differences in soil mineral N residual among treatments at *p* < 0.05.
