# Supplementary material for: Enhancing Soybean and Maize Yields through Improved Nitrogen and Soil Water Use Efficiencies: A 40-Year Study on the Impact of Farmyard Manure Amendment in Northeast China
Source: Plants (Basel). 2024 Feb 10;13(4):500. doi: 10.3390/plants13040500 (PMC10893496; doi:10.3390/plants13040500)
Supplement: Supplementary file 1 [file plants-13-00500-s001.zip › plants-2828610-supplementary.pdf]

Supplementary Materials

Supplementary Figures

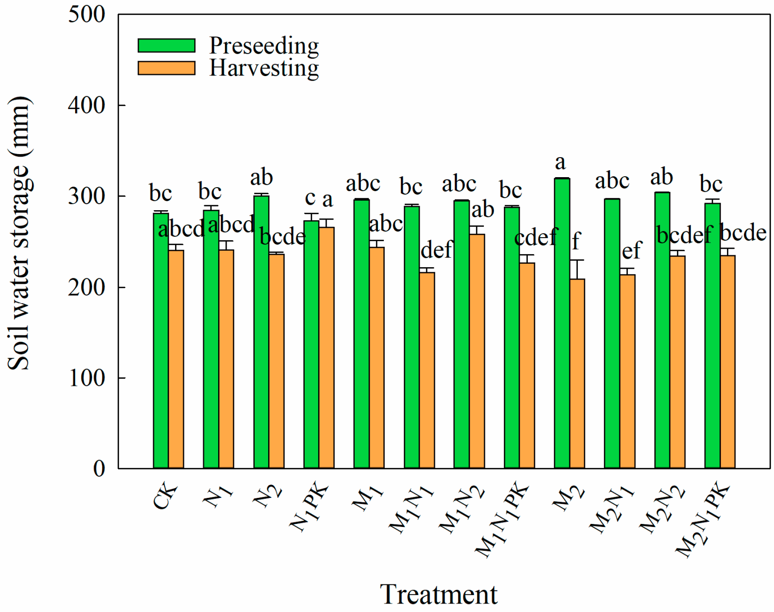

Figure S1. Soil water storage in the 0-100 cm soil layer before soybean sowing and after harvest in 2017. Different lowercase letters indicate significant differences in soil water storage in the 0-100 cm layers among treatments at $P < 0.05$.

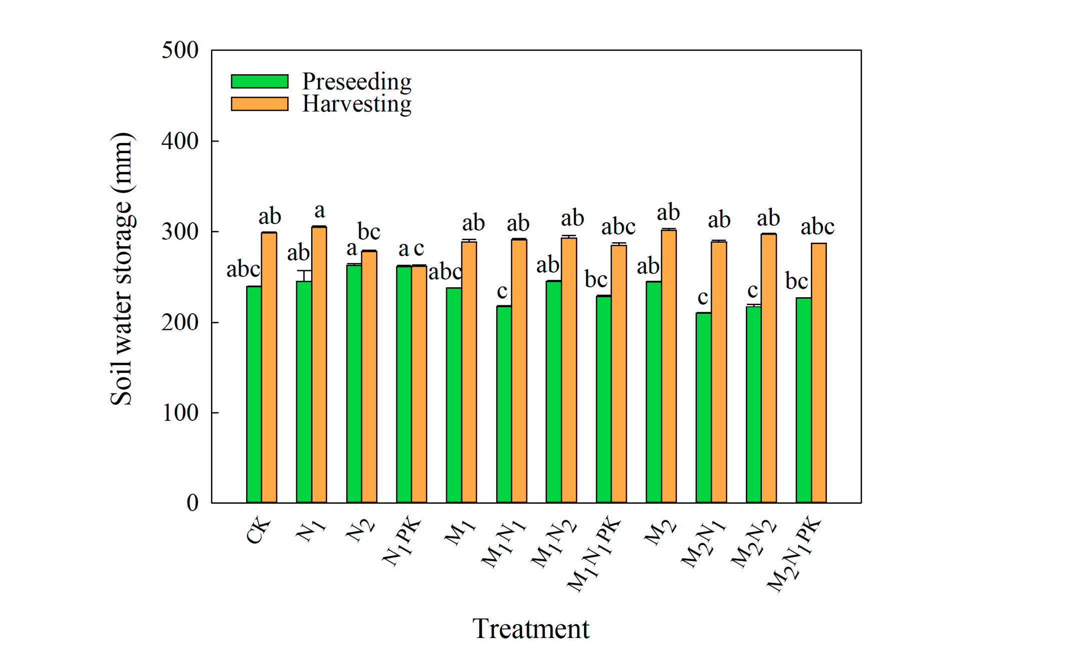

Figure S2. Soil water storage in the 0-100 cm soil layer before maize sowing and after harvest in 2018. Different lowercase letters indicate significant differences in soil water storage in the 0-100 cm layers among treatments at $P < 0.05$.

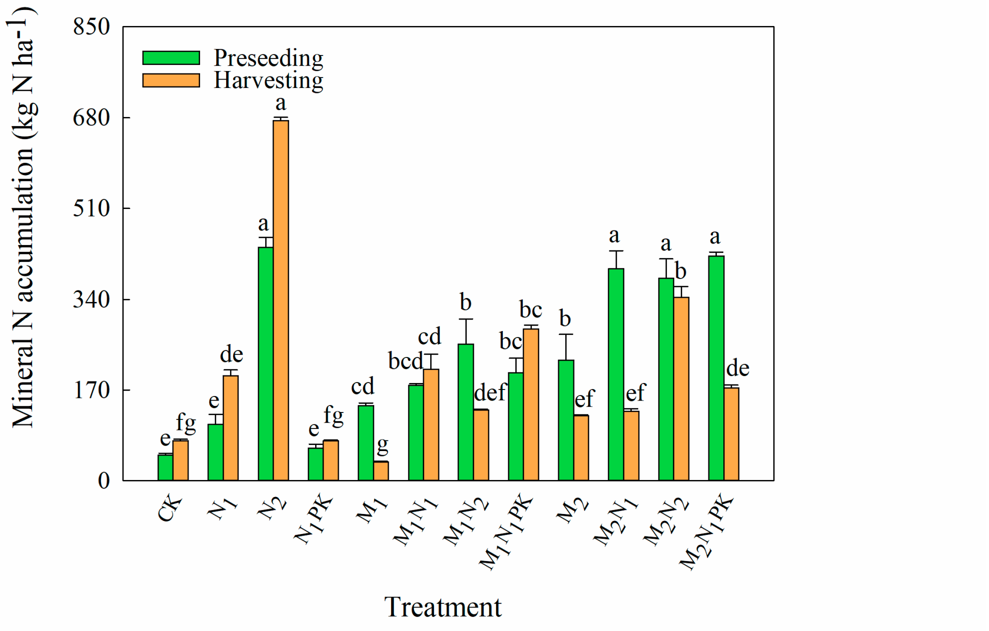

Figure S3. Mineral N residual in the 0-100 cm soil layer before soybean sowing and after harvest in 2017. Different lowercase letters indicate significant differences in soil mineral N residual among treatments at $P < 0.05$.

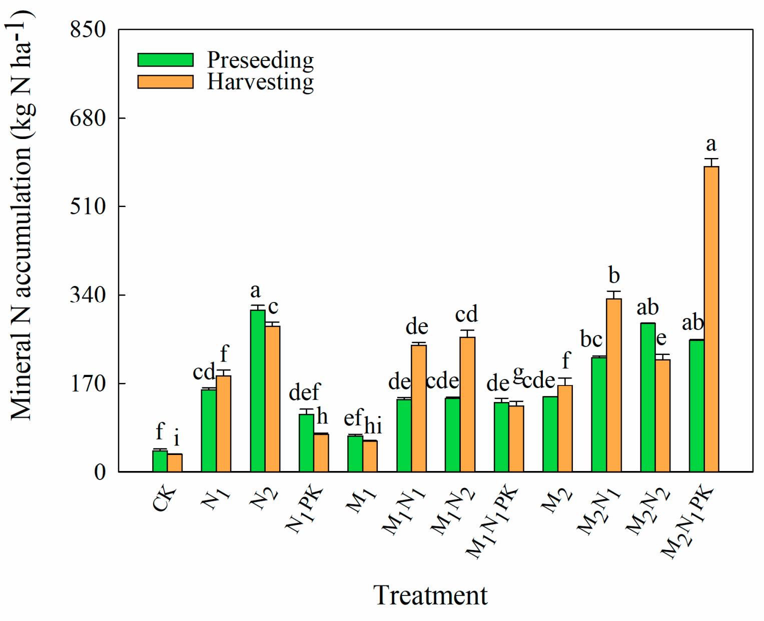

Figure S4. Mineral N residual in the 0-100 cm soil layer before maize sowing and after harvest in 2018. Different lowercase letters indicate significant differences in soil mineral N residual among treatments at $P < 0.05$.
